# Reviews and Bibliographical Notices

**Published:** 1882-03

**Authors:** 


### REVIEWS AND BIBLIOGRAPHICAL NOTICES.

A STUDY OF THE TUMORS OF THE BLADDER, with original contributions and drawings, BY ALEX. W. STEIN, M. D., Surgeon to Charity Hospital; Prof. of visceral anatomy and physiology at N. Y. College of Dentistry, etc., etc.—New York, Wm. Wood Co., 1881.

The subject of intra-vesical growths seems at first sight such a limited one, that it excites some surprise to find that a monograph of nearly a hundred pages could be written upon it, and what is more to the purpose—read with profit. Professor Stein shows, however, in this volume, that a much larger book could be written upon the subject by one having a less faculty for condensation than the author. The bibliography prefixed to the work occupies five pages, and covers the period from 1747 to 1881. Four cases occurring under the observation of the author are reported in addition to the very considerable number collated from the literature. The book is a valuable contribution to the study of the surgical diseases of the bladder, and the only complete monograph on neoplasms of this viscus.

The publishers' work is well done.

TRANCE AND MUSCLE READING, by George M. Beard, A. M., M. D.. etc., New York, 1882. Pp 40.

In this *brochure*, Dr. Beard gives a clear summary of the facts known about the state of trance, muscle-reading, and allied nervous phenomena. From the prominent connection of Dr. Beard with these subjects, the paper is of necessity largely polemical. It can, however, so far as we are able to judge, be relied on as a fair exposition of the differences between the author and other psychologists upon this much debated subject.

*Transactions of the Illinois State Dental Society for 1881.*

The welcome volume of transactions of the Illinois State Dental Society is at hand—better late than never. It should be possible to hand it around a little earlier. The delay from May to January is a little too long.

The papers, which are here given us maintain the reputation of the society, as being one of the most active, energetic and progressive of which our specialty may boast.

With one or two exceptions, the papers read were ably written, upon topics of genuine interest and real merit. Dr. Dean's paper on the "Development of Enamel," Dr. Harlan's on the "Characteristics of Saliva in Syphilitic Persons" and "Fractures of the Inferior Maxilla" by Dr. Gilmer, are all worthy of careful reading.

The Society will hold it next annual session at Quincy, May, 1882.

*Le Progres Dentaire*—This French journal has just completed its sixth volume. It has for its aim and object the reviewing of dental and medical exchanges, thus gleaning, and translating into French all the most valuable matter, and presenting it to its French readers in the most condensed and available form. *Le Progres* is now giving in detail the work of dental education (" Reform " a l' Anglaise) and obtaining of legal recognition, to which, as a matter of course, there is much opposition on the part of quite a large body of dentists. We wish success to *Le Progres*, as well as to "Reform."

*The New England Medical Monthly*, edited and published by Dr. Wm. C. Wile, Sandy Hook, Ct., is a neat, well-filled journal, which has reached the fifth number of volume 1. The monthly is well edited and deserves success.

*The Missouri Valley Medical Journal* is a new monthly hailing from one of the "future great cities of the West," St. Joseph, Mo. It is edited by Drs. W. C. Boteler and F. C. Hoyt, with a staff of five editors. The first number presents a promising appearance.

*The American Journal of Obstetrics, and Diseases of Women and Children,* which we take pleasure in adding to our exchange list, is one of the most valuable and practical journals published, and should be on the table of every practitioner. A monthly supplement, containing reports of society proceedings, and abstracts of articles in current foreign literature was begun in January.

*The Odontographic Journal* is a neat quarterly, devoted to dentistry, published by Davis & Leyden, Rochester, New York. Dr. J. Edward Line is the editor.

*Report of the Bureau of General Sanitary Science, Climatology and Hygiene to the American Institute of Homeopathy.* Reprint from Transactions American Institute of Homeopathy, Pittsburg, 1881. Pp. 117.

*Fourth Annual Report of the Presbyterian Eye and Ear Charity Hospital,* Baltimore, 1882. Pp. 35.

The report of the surgeon, Dr. J. J. Chisolm, shows that 3,145 cases were treated at this institution during the twelve months from December 1880 to December 1881.

*Diseases of the Ear in Locomotive and other Engineers, Firemen and Conductors, which may endanger the lives of the Traveling Public,* by Laurence Turnbull, M. D. etc. Reprint from Trans. Med. Soc. of Penna. Pp. 6.

*Case of a Pistol-shot wound of the second and third cervical vertibræ, considered in its Medico-Legal aspects—Attempted suicide—Death,* by Edward C. Harwood, M. D., etc. Reprint from Bulletin of the Medico-Legal Society, of New York, March, 1881. Pp. 12.

*Anæsthetics Medico-Legally considered,* by J. G. Johnson, M. D. Reprint from the Bulletin of the Medico-Legal Society, of New York, December, 1881. Pp. 23.

A very excellent paper, giving a concise summary of recent cases in medical jurisprudence, resulting from the administration of anæsthetics. The author particularly enforces the precept, never to give an anæsthetic to a woman, except in the presence of a third party, who should also, by preference, be a woman.

*The New England Journal of Dentistry and Allied Sciences,* makes a pretty bow with its first number. It is filled with interesting reading matter and is edited with good taste and judgment. Its home is in Springfield, Mass.

